# Supplementary material for: RAG1 and RAG2 non-core regions are implicated in leukemogenesis and off-target V(D)J recombination in BCR-ABL1-driven B-cell lineage lymphoblastic leukemia
Source: eLife. 2024 Jul 26;12:RP91030. doi: 10.7554/eLife.91030 (PMC11281782; doi:10.7554/eLife.91030)

Figure 2—figure supplement 1—source data 3

Original file for the PCR analysis in Figure 2—figure supplement 1B

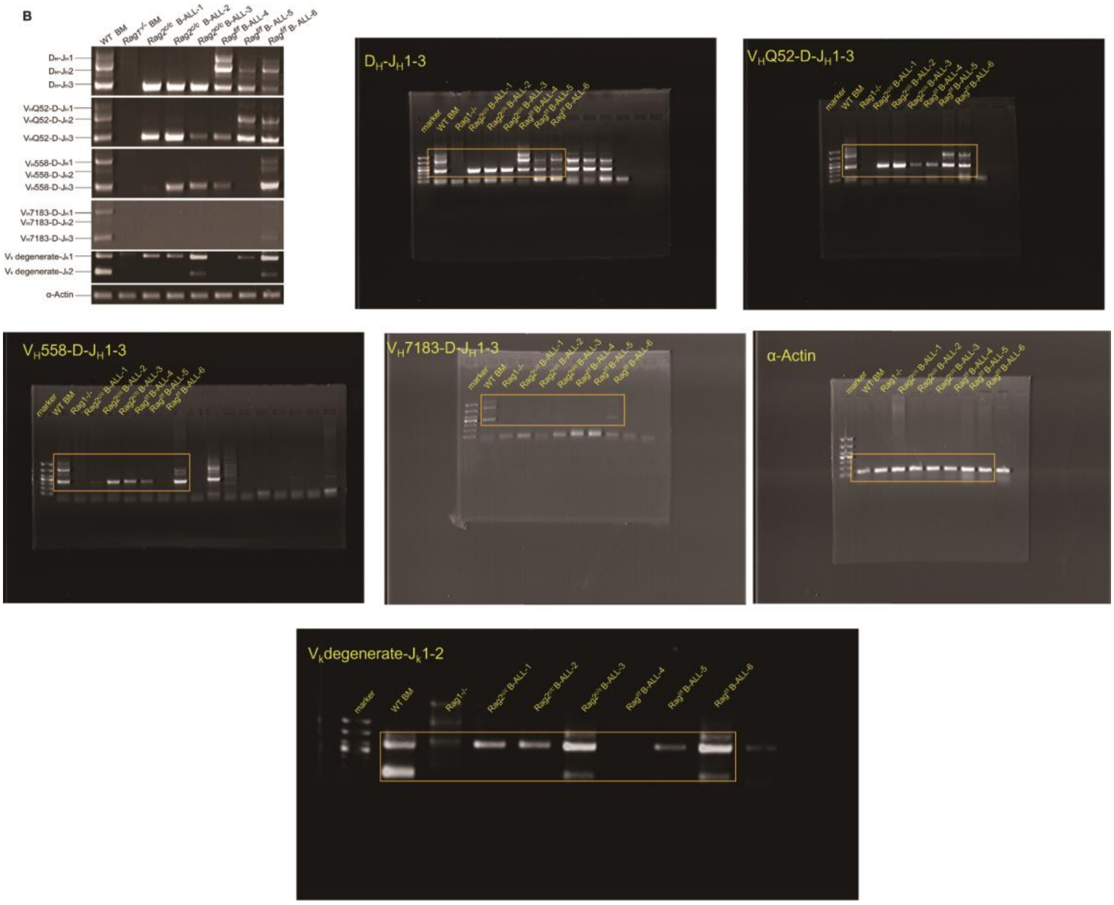

Supplement: Figure 2—figure supplement 1—source data 2. [file elife-91030-fig2-figsupp1-data2.pdf]
